# In silico functional and structural characterization revealed virulent proteins of *Francisella tularensis* strain SCHU4 

**DOI:** 10.22099/mbrc.2022.43128.1719

**Published:** 2022-06

**Authors:** Prerna Goel, Tanya Panchal, Nandini Kaushik, Ritika Chauhan, Sandeep Saini, Vartika Ahuja, Chander Jyoti Thakur

**Affiliations:** 1 Department of Bioinformatics, Goswami Ganesh Dutta Sanatan Dharma College, Sector 32 C, Chandigarh, India; 2 Department of Biophysics, Panjab University, Sector 25, 160014, Chandigarh, India

**Keywords:** Domains, Drug target, Virulence factor, Annotation, Microbial resistance, Bioinformatics

## Abstract

*Francisella tularensis* is a pathogenic, aerobic gram-negative coccobacillus bacterium. It is the causative agent of tularemia, a rare infectious disease that can attack skin, lungs, eyes, and lymph nodes. The genome of *F. tularensis* has been sequenced, and ~16% of the proteome is still uncharacterized. Characterizations of these proteins are essential to find new drug targets for better therapeutics. *In silico* characterization of proteins has become an extremely important approach to determine the functionality of proteins as experimental functional elucidation is unable to keep pace with the current growth of the sequence database. Initially, we have annotated 577 Hypothetical Proteins (HPs) of *F. tularensis *strain SCHU4 with seven bioinformatics tools which characterized them based on the family, domain and motif. Out of 577 HPs, 119 HPs were annotated by five or more tools and are further screened to predict their virulence properties, subcellular localization, transmembrane helices as well as physicochemical parameters. VirulentPred predicted 66 HPs out of 119 as virulent. These virulent proteins were annotated to find the interacting partner using STRING, and proteins with high confidence interaction scores were used to predict their 3D structures using Phyre2. The three virulent proteins Q5NH99 (phosphoserine phosphatase), Q5NG42 (Cystathionine beta-synthase) and Q5NG83 (Rrf2-type helix turn helix domain) were predicted to involve in modulation of cytoskeletal and innate immunity of host, H2S (hydrogen sulfide) based antibiotic tolerance and nitrite and iron metabolism of bacteria. The above predicted virulent proteins can serve as novel drug targets in the era of antibiotic resistance.

## INTRODUCTION

*Francisella tularensis,* which was first accidentally discovered in 1911, is the causative agent of tularemia* and *is considered a dangerous potential biological weapon. This bacterium is a small facultative, aerobic, non-spore-forming, non-motile intracellular pathogen that mainly replicates in macrophages [1]. The pneumonic form of tularemia is life-threatening and can result in death. According to the Centers for Disease Control and Prevention (CDC), *F.tularensis *is a viable biological warfare agent due to its high infectivity, ability to cause death, and low infectious dose [2]. The family *Francisellaceae* includes three species in the genus *Francisella* and four subspecies of *F. tularensis*. Although all have been associated with human disease, only the *tularensis* and *holarctica* subspecies of *F. tularensis* are relatively common [3]. *F. tularensis* is covered by a capsule that is required for virulence [4,5]. Recently, the presence of Pili (type 4) has been observed which may be the cause of the virulence of the bacteria [6]. *F. tularensis* has been reported in birds, reptiles, fish, invertebrates, and mammals, including humans. It is caused by contact with infected animals or vectors such as ticks, mosquitoes, and deer flies which makes it a dangerous and highly infectious agent for biological warfare [7-10]

The genome of *F. tularensis *strain SCHU4 has been sequenced. Its 1.89 Mb base pair long genome with 1910 total genes is contained in only one chromosome. Approximately 83% of the genome is coding which codes about 1667 proteins. Out of these coding proteins, 577 (~16%) are hypothetical proteins (HPs), i.e., there is no experimental evidence regarding the proteins' function and structure, implying that these proteins are yet to be annotated [11, 12] 

Due to the advancements in sequencing technologies, a large amount of protein sequence data is generated that needs to be annotated or characterized but still a significant proportion is demarcated as hypothetical. To keep pace with the exponential growth of sequence data, computational approaches play an important role in deciphering the function of HPs that reduce the laborious experimental protocols [13, 14]. These approaches are important for understanding species-specific pathogenicity, new target identification, pathway associations, and evolutionary processes [15-17].

In-silico approach has been successfully applied previously to various species like *Mycobacterium tuberculosis, Streptococcus Gordonii, Vibrio cholera, Staphylococcus aureus, Haemophilus influenza, Shigella flexneri, Exiguobacterium antarcticum* strain B7, *Neisseria gonorrhoeae* and *Chloroflexus aurantiacs *[18-26].

Hence, considering the bacterium's pathogenicity and the need to discover a new drug target in the era of antimicrobial resistance, we have computationally annotated uncharacterized proteins of *F. tularensis *strain SCHU4 using various bioinformatics tools. The study also identified the most virulent proteins from 577 HPs that can serve as novel drug targets. 

## MATERIALS AND METHODS

**Figure 1:**
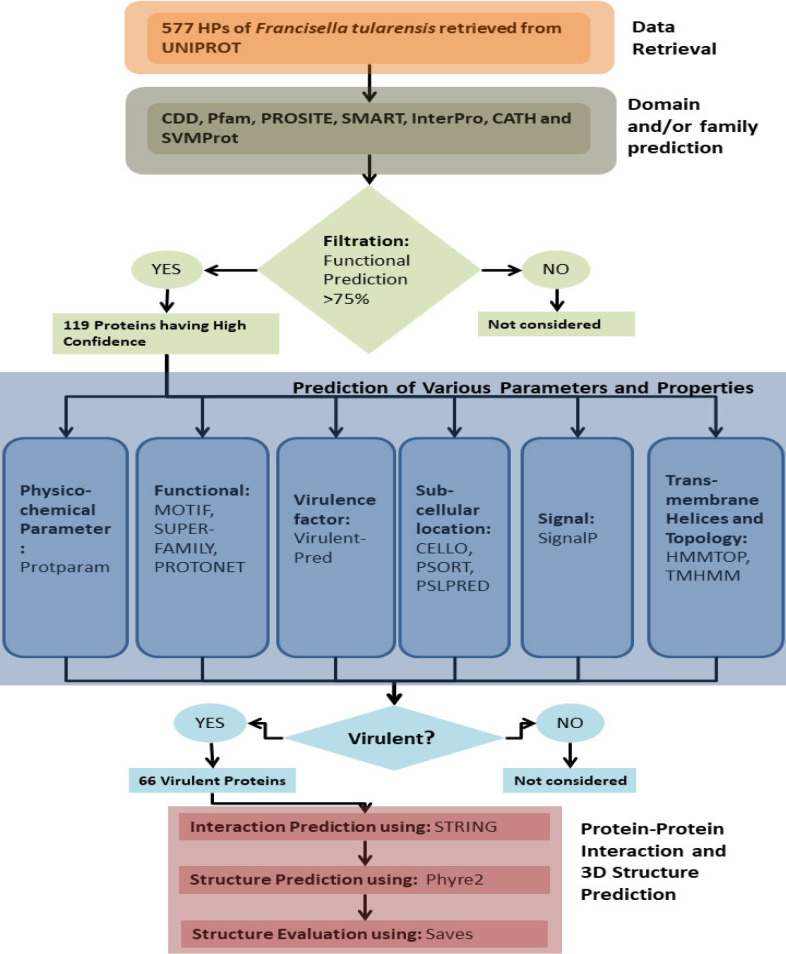
Flowchart of Methodology

**Data Retrieval: **In this study, hypothetical proteins of *F. tularensis *strain SCHU4 were retrieved from UniprotKB (https://www.uniprot.org/) [27]. Out of the total 3,733 proteins of *F. tularensis, *577 were uncharacterized proteins. The computational workflow used for this study has been illustrated in Figure 1.

**Functional annotation and filtration of HPs: **To assign biological function to HPs is a major obstacle and a challenging task, we have used various databases and tools for functional annotation including CDD (Conserved Domain Database) (https://www.ncbi.nlm.nih.gov/ Structure/cdd/wrpsb.cgi) [28], SMART (Simple Modular Architecture Research Tool) (http:// smart.embl-heidelberg.de/) [29] CATH (Class, Architecture, Topology, Homology) (https:// www.cathdb.info/) [30], PROSITE (https://prosite.expasy.org/) [31], Pfam (http://pfam. xfam.org/) [32], InterPro (https://www.ebi.ac.uk/interpro/) [33] and SVMProt (https://bio.tools/ svm-prot) [34]. CDD, SMART, and PROSITE were used for identification of conserved domains, and CATH was used to classify the domains within a structural hierarchy. Pfam, SVMProt, and InterPro were used to classify the HPs into functional families based on similarity. We have selected only those HPs for further analyses, which were annotated by five or more tools out of seven (i.e.75% annotation criteria,). To validate our initial annotation result we have further used MOTIF (https://www.genome.jp/tools/motif/) [35], SUPERFAMILY (https://supfam.mrc-lmb.cam.ac.uk/SUPERFAMILY/) [36], PROTONET (http://www.protonet. cs.huji.ac.il/) [37] to classify the HPs into functional families. The MOTIF assigns function to the HPs by aligning with the profile generated from multiple sequence alignment while ProtoNet is based automated hierarchical clustering that rely on all against all BLAST (Basic Local Alignment Search Tool) similar search and map HPs into an existing clustering and SUPERFAMILY predict domain in HPs based Structural Classification of Proteins (SCOP) database hierarchy at superfamily level that groups together the most distantly related domains [35-37]. Details of analysis were given in supplementary Tables S1 and S2.

**Prediction of physicochemical parameters and subcellular location: **For physiochemical parameters prediction ProtParam (https://web.expasy.org/protparam/) was used. We retrieved various physicochemical properties of the protein parameters i.e. .molecular mass, theoretical isoelectric point (pI), amino acid composition, atomic composition, extinction coefficient, instability index, aliphatic index, and grand average hydropathy (GRAVY) [38].

To determine the subcellular localization we used three predictors; CELLO, PSLpred, and PSORTb. CELLO (subcellular LOcalization predictor) (http://cello.life.nctu.edu.tw/), a two-level support vector machine to predict the subcellular locations [39]. On the other hand, PSLpred (https://webs.iiitd.edu.in/raghava/pslpred/submit.html) relies on SVM method [40] and PSORTb (https://www.psort.org/psortb/) combine experimental and computational prediction for prediction of subcellular locations of HPs [41]. TMHMM (http://www.cbs.dtu.dk/services/ TMHMM/) [42], HMMTOP (http://www.enzim.hu/hmmtop/html/submit.html) [43] and SignalP (http://www.cbs.dtu.dk/services/SignalP/) [44] were used to determine the transmembrane helices and signal peptide in HPs.

**Prediction of virulence factor: **Virulence factor analysis was done by VirulentPred (http://203.92.44.117/virulent/submit.html), a SVM-based tool that calculates potential virulent proteins and use five-fold cross-validation technique. We have selected default threshold value i.e. 0.0 and score from 0.0 to 1 predict the protein as virulent and minus score indicate non-virulent protein [45].

**Protein-protein interaction prediction and tertiary structure prediction: **STRING (Search Tool for the Retrieval of Interacting Genes/Proteins) (https://string-db.org/) database was used to predict the protein-protein interactions of the virulent HPs [46]. Among 66 HPs, we have selected only those proteins which were associated with other known proteins with high confidence score. STRING provides a functional link between proteins called networks along with their scores.

Furthermore, we have determined the 3D structure of those proteins that possess interactions with known proteins with a high confidence score. To predict the protein structure, which is more conserved than protein sequence, we use a protein homology/analogy recognition engine (Phyre 2) (http://www.sbg.bio.ic.ac.uk/phyre2/html/page.cgi?id=index) that gather homologous sequences against query protein and then scanned against HMM database of known protein structure [47, 48]. The structure evaluation was performed by Procheck using SAVES v6.0 server (https://saves.mbi.ucla.edu/) that analyzes the number of residues falling in the allowed region in the Ramachandran Plot as a measure of quality [49].

## RESULTS

**Figure 2:**
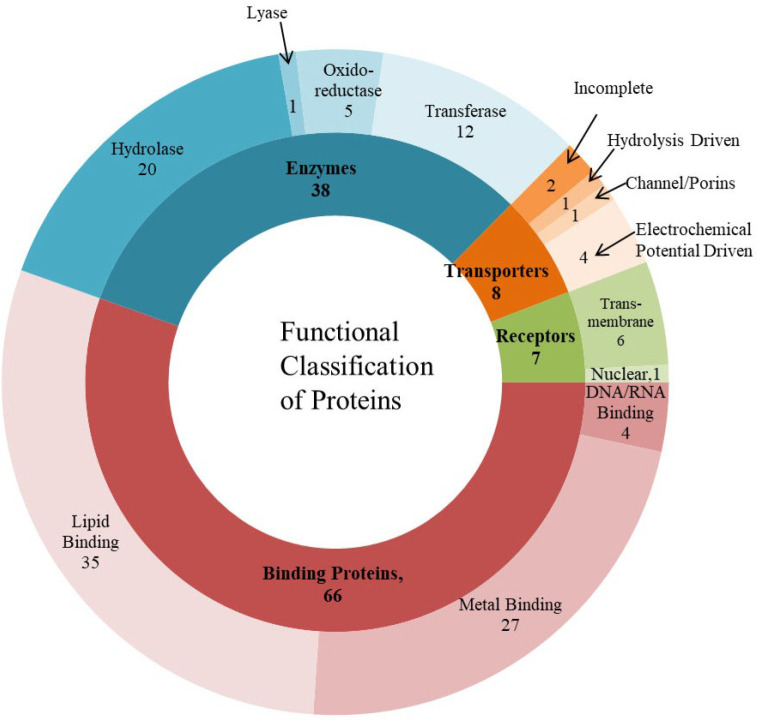
119 HPs classified into enzymes (38), binding proteins (66), receptors (7) and transport proteins (8)

We have extensively analyzed 577 HPs of F. tularensis using various bioinformatics tools, i.e., CDD, Pfam, SMART, PROSITE, InterPro, CATH, and SVMProt, for domain and/or functional prediction. If the same function is assigned for HPs by five or more tools, then only those HPs were selected for further annotation. Using this strategy, we have identified 119 HPs out of 577. These 119 HPs are further analyzed by using Motif, Superfamily, and Protonet for protein family characterization and were classified as 38 enzymes, 66 binding proteins, 7 receptors, 8 transport proteins as given in Fig. 2 and supplementary Table S2.

Protoparam was used for the prediction of various physiochemical parameters of the filtered 119 proteins. These parameters were molecular weight, aliphatic index, instability index, theoretical pI, etc. >40. The instability index of the protein signifies that the protein is unstable. We identified 28 proteins whose instability index values were greater than 40. The theoretical pI of the HPs lies between 10 and 4. The GRAVY values indicate both hydrophobic (negative) and hydrophilic (positive) HPs [50-52]. The Aliphatic Index of the HPs varied between 160 and 59. The physicochemical parameters of 119 HPs are listed in supplementary Table S3. 

Subcellular location correlates protein biological function as cell offer different chemical environment and interaction [53]. Signal peptides control transport of proteins to target location in all organisms [54, 55]. Membrane proteins are involve in signaling between cells, transport and energy transduction [42, 43]. Based on the average subcellular prediction results of the three tools HPs were located cytoplasm (40%), membrane (39%), extracellular region (18%), and periplasm (3%). We have predicted presence of signal peptide sequence in 78 HPs. HMMTOP and TMHMM server predicted 65 and 61 proteins as transmembrane helices. These prediction results help to better understanding the new drug target and detail results are mentioned in supplementary Table S4.

Prediction of virulent protein is important for identification of novel targets and understanding the complex virulence mechanism of pathogen [45]. VirulentPred predicted 66 HPs as virulent with the score ranges from 0.95 to 1 which is a higher score that minimize false positive hits (supplementary Table S5).

**Figure 3:**
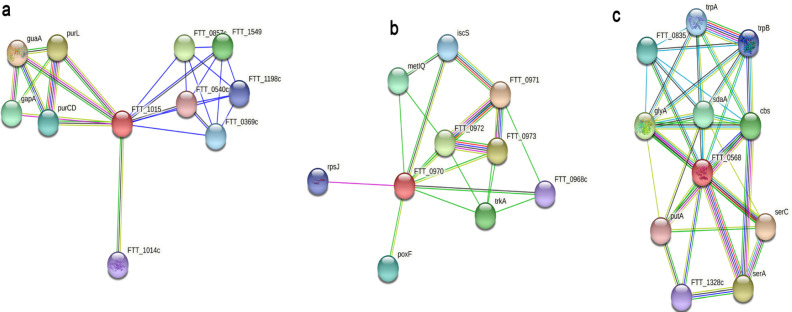
Protein-protein Interaction Network of Q5NG42, Q5NG83 and Q5NH99 proteins by STRING database

Protein-protein interaction network: We have predicted the association of 66 HPs with other proteins that can further elaborate the role of HPs. Among the complete set of 66 proteins, 25 were dropped out as these did not show any significant interactions. The remaining 41 proteins are related to the known genes with high confidence. We identified interacting partners involved in lipid biosynthesis, signal transduction, transcriptional regulators, iron sulfur binding, extracellular signals, and stimuli. Some proteins interacted with the type 4 pilli, which is suspected to be the cause of the pathogenicity of *F. tularensis *[6]. Our study found that out of 41 proteins, three proteins Q5NH99, Q5NG42, and Q5NG83, had a very high confidence score of 98%, 95%, and 89.5%, respectively, with their interacting partner. Q5NG83 interacts with the ABC Transporter enzyme, which plays a role in bacterial virulence mainly associated with the uptake of nutrients, while Q5NG42 interacts with GMP synthase required for virulence factor production and Q5NH99 interact with proteins involved in metabolism [56, 57]. The STRING results are shown in Figure 3.

**Figure 4:**
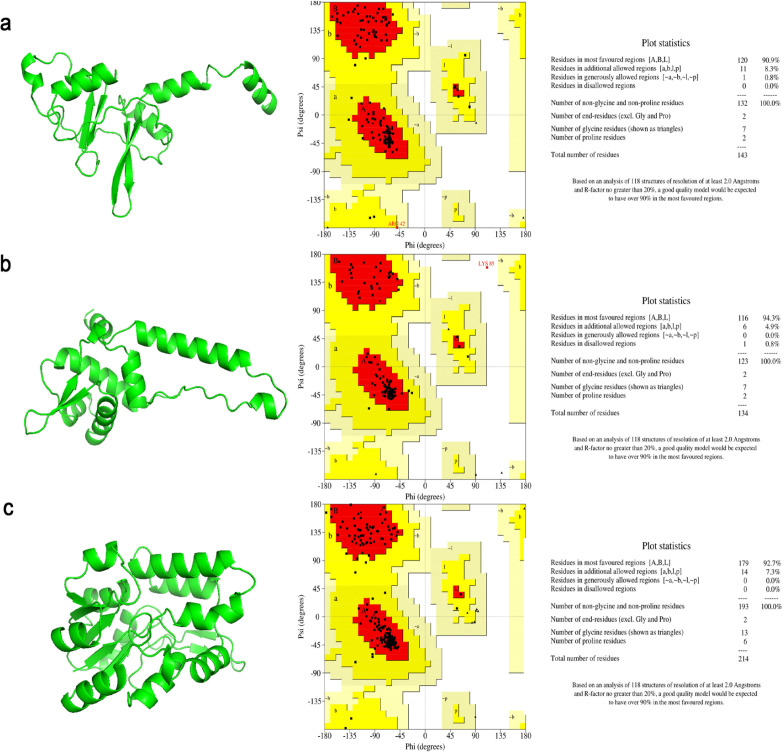
Three-Dimensional structural prediction of (a) Q5NG42, (b) Q5NG83 and (c) Q5NH99 proteins by Phyre2 and evaluation of structure by Ramachandran plot by SAVES which show score above 90%

Phyre2, a web-based server to predict protein structure based on the principle of homology-based modeling. Query protein is scanned against HMMs proteins of known structure, and top-scoring alignments are used to construct crude backbone, and then model is corrected by loop modeling, and side-chain is added to generate the final model [47, 48]. We have successfully generated the three best models with high confidence i.e., Q5NG42 protein with 99.9% coverage and 70% confidence, Q5NG83 with 100% confidence and 98% coverage, Q5NH99 with 100% confidence and coverage. The structures predicted by using Phyre2 were evaluated by Procheck to check the quality of the 3D model and more than 90% residues were in favoured region of Ramachandran Plot for all the three predicted structure as shown in Figure 4.

## DISCUSSION

Cost-effective sequencing technology has made bacterial genome sequencing affordable to several labs world widely [58]. The published bacterial genome consists of a large group of hypothetical proteins which must be annotated to understand disease pathogenesis, virulent determinants, survival, and propagation. Characterization of HPs by various bioinformatics tools may identify potential biomarkers for the screening and diagnostics of diseases as well as pharmacological targets to design and discover novel drugs [59].

In the present study, we have annotated HPs of *F. tularensis* by various computational tools to predict the function and structure of proteins that can potentially be involved in the toxicity of the pathogen. The initial step involved identification of conserved region i.e. domain and motif which provide the clue about protein function. We started analysis of 577 HPs using seven functional tools and those HPs are selected for further analysis if the result of each HPs is predicted by five or more tools i.e.>75% prediction analysis which retrieves 119 HPs. These HPs were further annotated for various properties including physicochemical parameters like GRAVY index, aliphatic index, pI, Instability index, cellular localization, transmembrane analysis, functional analysis, and virulent prediction. We then predicted interacting partners of these 66 proteins and based on high confidence score we have selected 3 virulent proteins Q5NH99, Q5NG42 and Q5NG83 with 98%, 95% and 89.5% confidence score respectively (Table 1). 

**Q5NH99 **predicted as phosphoserine phosphatase (PSP), a key essential metabolic enzyme catalyse the dephosphorylation of phosphoserine to serine and inorganic phosphate and is a member of haloacid dehalogenase (HAD) superfamily. PSP have been demonstrated to facilitate entry of Porphyromonas gingivalis into host cells by modulating host cytoskeletal architecture, innate immune responses, dephosphorylating colicin and NF-κ [60-62].

**Table 1:** Functional and structural annotation results of virulent proteins: Three virulent proteins with their annotated function, virulence score (0.4 is considered as threshold), and Phyre2 structure prediction and evaluation score

**Protein ID**	**Annotated Function**	**VirulentPred Score**	**Final Prediction**	**Structure Modelling confidence score by Phyre2**	**Structure Evaluation (percent amino acid residues in most favoured region)**
Q5NH99	Phosphoserine phosphatase	1.0016	Virulent	98%	92.7%
Q5NG42	Cystathionine beta-synthase	0.9776	Virulent	95%	90.9%
Q5NG83	Rrf2-type helix turn helix domain	1.0957	Virulent	89.5%	94.3%

**Q5NG42**, a 196 amino acid length was predicted as Cystathioninebeta-synthase (CBS) catalyzes a condensation reaction to convert homocysteine to cystathionine. This enzyme also produces hydrogen sulfide (H2S) during this catalysis reaction [63]. A number of previous studies in bacterial species have revealed that production of H2S provides antibiotic tolerance and serve as universal defence mechanism [66-66]. Further supportive evidence was gained by study in which the inactivation of CBS leads to decrease in H2S level and hence more sensitivity towards antibiotics [67]. Hence, CBS can act as a main target against antibiotic resistance in era of antimicrobial resistance (AMR).

**Q5NG83 **Rrf2-type HTH domain is a 130 residues long DNA binding, helix-turn-helix domain. This domain is present in the proteins that serve as transcription regulators of rrf2 family. Usually, these transcription regulators regulate the genes of bacteria that are involved in nitrite and iron metabolism. For instance, in *Escherichia coli* one the regulator protein, nsR that contains Rrf2-type HTH domain regulates the NO (nitric oxide) sensitive transcription and thus protects the cell from the NO stress. Similar transcription regulator protein with same role was also found in other bacteria such as *Bacillus subtilis* and *Streptomyces coelicolor* whereas *Rhizobium leguminosarum* contains a transcription regulator protein rirA which is an iron-responsive regulator that involved in uptake of iron [68, 69].

Our study has predicted virulent proteins that can play a vital role in future therapeutics of tularemia. These results need to be analyzed in vitro to verify these results experimentally to check the feasibility of these proteins as possible drug targets and their feasibility. These proteins can be essential in determining the virulence mechanism as well as act as the keys for unlocking the mystery related to the immunity of *F. tularensis* to antibiotics. By further analysis of these proteins, we can also look for binding sites and try to dock leads to these proteins to develop a treatment that has a higher efficacy with fewer side effects. 

A recent study by Kumar et al. used pan-genome analysis and genome mining approach to explore the diversity of the genus Francisella. The whole-genome sequence comparisons of the 26 genomes in their study revealed average nucleotide identity (ANI) of 97.0–99.5% for the human pathogens strains cluster, whereas ANI values of 74.2–90.4% were found in the strains that are mainly related to water and environment [70].

Thus the above study shows that there exhibit a good degree of similarity in the coding regions of Francisella genomes. Hence, the functional characterization of HPs in our study may help in the study of proteins of other strains of Francisella

## Conflict of Interest

Authors have no conflict of interest.
